# POLICY SERIES: WHAT IS “GOOD WORK” IN LONG-TERM SERVICES AND SUPPORTS? SETTING A RESEARCH AND POLICY AGENDA FOR DIRECT CARE WORK

**DOI:** 10.1093/geroni/igae098.1985

**Published:** 2024-12-31

**Authors:** Jennifer Morgan, Philip Taylor, LIza Behrens

**Affiliations:** Chair; Co-Chair; Discussant

## Abstract

This symposium considers the importance of the care-work nexus in long-term care research and policy. Person-centered care delivery is widely accepted as delivering optimal client outcomes, but this is often not practiced. Job quality is considered a crucial determinant of well-being for individuals, society and of organizational performance, yet direct care work is not generally afforded “good work” job quality. Raising standards of appropriate care and care work job quality is essential to improve the life quality of both clients and workers. Drawing from empirical research in the USA and internationally, this session considers the current state of long-term care work across key dimensions of job quality. Critically from a job quality perspective, this session foregrounds the authentic worker voice in identifying a research and policy agenda focused on addressing the care-work nexus. First, we will provide an overview of job quality across long-term care services and supports sectors highlighting the target areas for research and policy advocacy by sector. Second, we will engage workers and union leaders, using a community-engaged co-design framework, in the local SEIU 775 Washington to react to the job quality domains and provide feedback on priority-setting. Third, Washington-based SEIU leaders will collaborate to review the policy, advocacy and negotiation wins made for clients and worker in conversation with employer partners. Finally, scholars, in collaboration with workers/leaders, will summarize the areas targeted for future research and policy agendas aimed at improving job quality and person-centered approaches for both workers and clients.

